# Vaccinating Children against COVID-19: Commentary and Mathematical Modeling

**DOI:** 10.1128/mbio.03789-21

**Published:** 2022-01-18

**Authors:** Michael T. Hawkes, Michael F. Good

**Affiliations:** a Department of Pediatrics, University of Alberta, Edmonton, Alberta, Canada; b Department of Medical Microbiology and Immunology, University of Alberta, Edmonton, Alberta, Canada; c School of Public Health, University of Alberta, Edmonton, Alberta, Canada; d Distinguished Researcher, Stollery Science Lab, University of Alberta, Edmonton, Alberta, Canada; e Member, Women and Children's Research Institute, University of Alberta, Edmonton, Alberta, Canada; f Institute for Glycomics, Griffith University, Gold Coast, Australia; g University of Alberta, Edmonton, Canada; Yale School of Public Health

**Keywords:** SARS-CoV-2, child, epidemiology, mRNA vaccine

## Abstract

With the recent licensure of mRNA vaccines against COVID-19 in the 5- to 11-year-old age group, the public health impact of a childhood immunization campaign is of interest. Using a mathematical epidemiological model, we project that childhood vaccination carries minimal risk and yields modest public health benefits. These include large relative reductions in child morbidity and mortality, although the absolute reduction is small because these events are rare. Furthermore, the model predicts “altruistic” absolute reductions in adult cases, hospitalizations, and mortality. However, vaccinating children to benefit adults should be considered from an ethical as well as a public health perspective. From a global health perspective, an additional ethical consideration is the justice of giving priority to children in high-income settings at low risk of severe disease while vaccines have not been made available to vulnerable adults in low-income settings.

Recently, Pfizer and BioNTech provided evidence that their mRNA vaccine against SARS-CoV-2 is safe and immunogenic in children 5 to 11 years of age (https://www.businesswire.com/news/home/20210920005452/en/Pfizer-and-BioNTech-Announce-Positive-Topline-Results-From-Pivotal-Trial-of-COVID-19-Vaccine-in-Children-5-to-11-Years). As countries prepare to incorporate school-age children into their vaccination schedules, the public health risks and benefits, as well as ethical considerations, should be carefully weighed.

One of the most distinguishing features of the SARS-CoV-2 pandemic is the dramatic correlation between age and disease severity. Early in the pandemic, a study in Switzerland from February to June 2020 demonstrated that the infection fatality rate for those over 65 years was 5.6 per 100 (1). This compared with a rate of 0.0016 per 100 in those aged 5 to 9 years and only 0.00032 per 100 for those aged 10 to 19 years (1). Data from the United States show that over 19 months, there were 349 deaths in those aged 0 to 17 years from a total of 606,389 deaths (2). Based on these figures, the chances of a child (<18 years old) dying from COVID-19 was ∼2,500 times less than that of an older American (>65 years old). Clearly, early on in the pandemic, although children were dying of COVID-19, it was at a very low rate. On the other hand, the emergence of the more infectious SARS-CoV-2 delta variant has led to increased cases in children, and there is increased recognition of pediatric morbidity associated with the multisystem inflammatory syndrome in children (MIS-C). Nonetheless, based on these figures, the benefits of vaccination are far greater for the elderly than for children, but there is a dynamic interplay of benefits and risks that needs to be considered.

Vaccines against SARS-CoV-2 are highly effective in clinical trials and real-world settings (3). Pfizer and BioNTech recently announced that their mRNA vaccine is safe and generates robust neutralizing antibody levels in children 5 to 11 years of age (https://www.businesswire.com/news/home/20210920005452/en/Pfizer-and-BioNTech-Announce-Positive-Topline-Results-From-Pivotal-Trial-of-COVID-19-Vaccine-in-Children-5-to-11-Years).

The public health benefits of vaccination are 2-fold, to protect the health of vaccinees and to contribute to herd immunity. Herd immunity occurs when the percentage of the population who are unable to transmit the virus as a result of immunity is sufficient to extinguish the epidemic. ℛ_0_ is the basic reproduction number for a virus in a nonimmune population. Immunity in the population occurs as a result of vaccination and natural immunity (as a result of infection). Using the number of “cases” as a proxy for the number of infections and, hence, the number with natural immunity, in the United States and the United Kingdom, approximately 10% of the population has natural immunity, whereas in Australia, 0.16% have natural immunity. However, these numbers are underestimates, as many people who have been infected remain asymptomatic and do not become cases (4).

If vaccinated individuals cannot transmit the virus, then the fraction of the population that needs to be vaccinated to end the epidemic is approximately 1 − 1/ℛ_0_. The ℛ_0_ for the dominant delta strain of SARS-CoV-2 has been reported to be between 5 and 8 (5). This means that between 80% and 87.5% of the entire population needs to be immune and nontransmitting. Even if ∼10% of the population were to develop immunity as a result of natural infection, then still between 70 and 80% of the population will need to be vaccinated and nontransmitting. There will be a need to vaccinate children of all ages, as well as adults, to reach 70 to 80%. Furthermore, recent reports show that the risk of household transmission is reduced by only ∼50% among vaccinated people who develop breakthrough SARS-CoV-2 infection (6).

While herd immunity is unlikely to be attainable through vaccination, higher levels of immunity will reduce the spread. Combined with social distancing and wearing of masks, we will be able to control focal epidemics (7).

To hasten and enhance the development of herd immunity, vaccination of children 5 to 11 years of age may be contemplated (∼10% of the entire population). However, vaccinating young children will have limited direct benefit to them as outlined above, but this population, by being immune, will protect the older and more vulnerable and, in particular, the nonvaccinated.

We hypothesize that childhood vaccination against SARS-CoV-2 will be associated with reductions in disease burden in children (directly, through disease attenuation among vaccinees) and in adults (indirectly, through herd immunity). We use mathematical modeling to support this hypothesis and provide quantitative estimates of the public health effects of childhood vaccination over 1 year in two jurisdictions, Australia and Alberta (Canada).

## RESULTS

**FIG 1:**
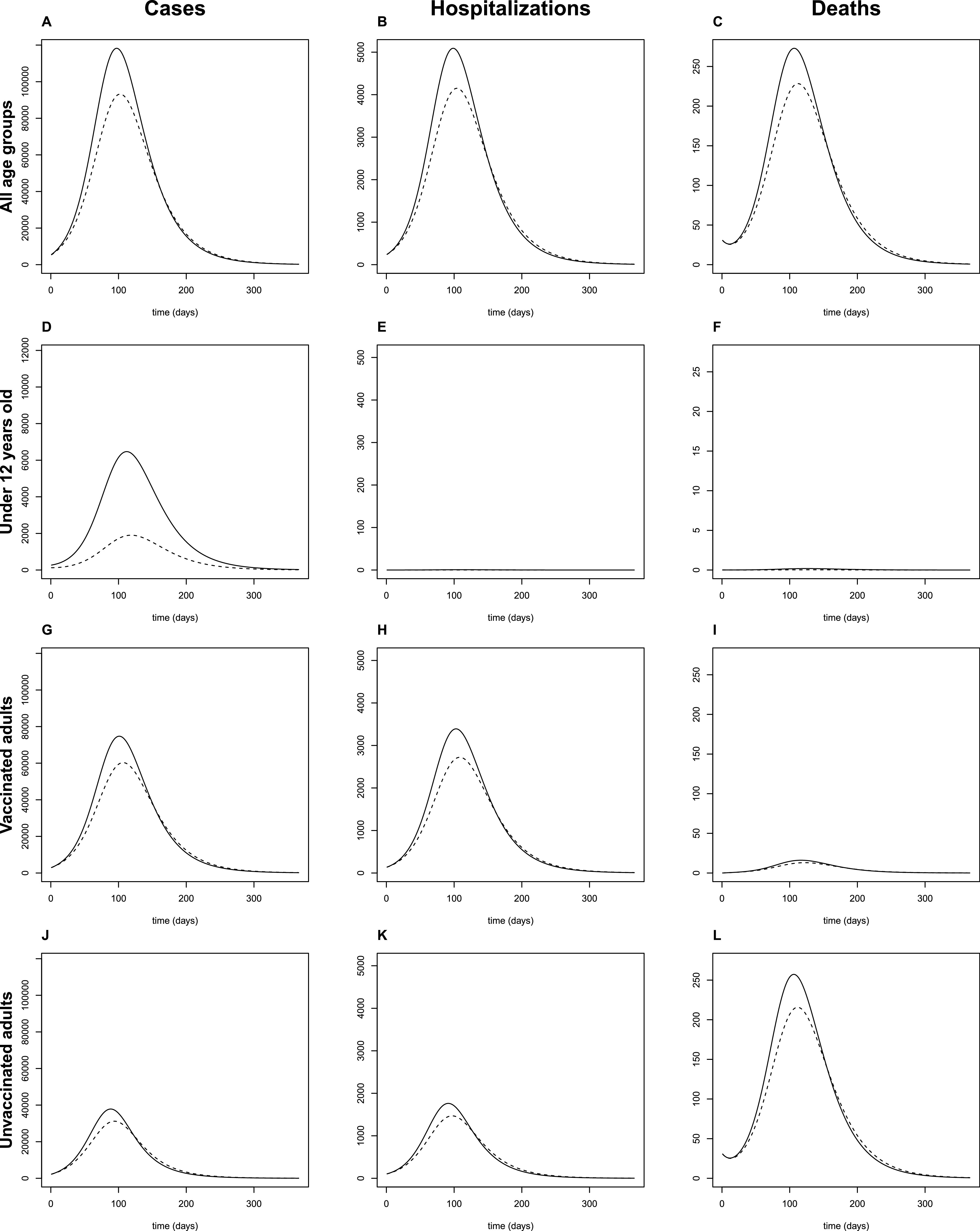
Projected wave of SARS-CoV-2 in Australia without (solid lines) and with (dashed lines) childhood vaccination (80% of children under 12 years of age). Using the SIR model, the epidemic curve was modeled over 1 year. (A to L) Results for the population (all age groups) are shown (A to C) and subdivided according to age and vaccine classes as follows: children under 12 (D to F), vaccinated adults (G to I), and unvaccinated adults (J to L). Model outcomes included daily incident cases (A, D, G, and J), hospitalizations (B, E, H, and K), and deaths (C, F, I, and L). The expected waves of cases, hospitalizations, and deaths was reflected in the model, with modest reductions associated with childhood vaccination. Hospitalizations and deaths were infrequent in children under 12.

We modeled the course of SARS-CoV-2 delta variant (ℛ_0_ = 5.08) in Australia (Table 1) and Alberta (Table 2) for 1 year, with and without childhood vaccination. The expected epidemic curve was observed, with infections eventually extinguishing to zero (Fig. 1).

**TABLE 1:** Simulation for Australia (ℛ_0_ = 5.08, 80% of adults vaccinated), including projected differences in cases, hospitalizations, deaths due to COVID-19, multisystem inflammatory syndrome in children (MIS-C), and vaccine adverse events associated with childhood vaccination^*c*^

Parameter	No. of patients with childhood vaccination	No. of patients with childhood vaccination (80% coverage)	No. of patients with absolute reduction	Relative reduction (%)
Cases of COVID-19 (×1,000)				
All age groups	12,200 (3,790 to 18,200)	10,500 (1,610 to 17,400)	1,760 (845 to 2,560)	14 (4.6 to 59)
5–11 yrs old	729 (262 to 1,030)	233 (25.9 to 564)	496 (241 to 563)	68 (45 to 91)
Vaccinated adults	7,800 (1,440 to 13,000)	6,860 (635 to 12,700)	932 (258 to 1,440)	12 (2 to 58)
Unvaccinated adults	3,530 (1,820 to 3,950)	3,220 (850 to 3,850)	305 (104 to 993)	8.6 (2.6 to 54)
Hospitalizations^*a*^				
All age groups	532,000 (152,000 to 857,000)	472,000 (68,800 to 834,000)	60,600 (23,200 to 97,700)	11 (2.7 to 56)
5–11 yrs old	78.4 (28.2 to 110)	25.1 (2.78 to 60.6)	53.3 (25.9 to 60.5)	68 (45 to 91)
Vaccinated adults	360,000 (62,700 to 650,000)	314000 (27,700 to 630,000)	46,500 (17,700 to 65,800)	13 (2.9 to 58)
Unvaccinated adults	172000 (79,000 to 208,000)	158,000 (37,700 to 204,000)	14,000 (3,860 to 42,500)	8.2 (1.9 to 53)
Deaths^*a*^				
All age groups	30,100 (12,900 to 39,000)	27,200 (6,230 to 38,000)	2,870 (1,020 to 6,740)	9.6 (2.6 to 52)
5–11 yrs old	22.0 (7.79 to 31.0)	3.53 (0.589 to 6.24)	18.5 (7.19 to 24.8)	84 (80 to 93)
Vaccinated adults	1,830 (289 to 3680)	1,590 (133 to 3,560)	241 (103 to 333)	13 (3.3 to 58)
Unvaccinated adults	28,200 (12,300 to 35,600)	25,600 (6,060 to 34,800)	2,610 (893 to 6,500)	9.3 (2.5 to 51)
MIS-C cases (0–19 yrs old)	230 (82.9 to 325)	73.7 (8.19 to 178)	157 (76.1 to 178)	68 (45 to 91)
Vaccine-related adverse events				
Myocarditis	20 (9.0 to 123)	38 (18 to 237)	−18 (−3 to −112)^*b*^	−93 (−15 to −570)^*b*^
Anaphylaxis	22 (9.8 to 35)	42 (19 to 68)	−20 (−8.1 to −50)^*b*^	−92 (−37 to −230)^*b*^

a Due to acute COVID-19.

b Negative sign indicates increase in cases with vaccination.

c Numbers in the table represent estimate (95% confidence interval).

**TABLE 2:** Simulation for Alberta (ℛ_0_ = 5.08), including projected differences in cases, hospitalizations, deaths due to COVID-19, multisystem inflammatory syndrome in children (MIS-C), and vaccine adverse events associated with childhood vaccination^*c*^

Parameter	No. of patients with childhood vaccination	No. of patients with childhood vaccination (80% coverage)	No. of patients with absolute reduction	Relative reduction (%)
Cases of COVID-19 (×1,000)				
All age groups	1,950 (788 to 2,870)	1,750 (595 to 2,760)	206 (114 to 237)	11 (4 to 25)
5–11 yrs old	97.3 (45.8 to 140)	34.6 (9.16 to 78.1)	62.7 (36.7 to 72.7)	64 (43 to 82)
Vaccinated adults	1,190 (284 to 1,990)	1,090 (217 to 1,950)	104 (35.5 to 129)	8.7 (1.9 to 24)
Unvaccinated adults	608 (405 to 667)	575 (327 to 654)	33.3 (12.6 to 76.8)	5.5 (1.9 to 19)
Hospitalizations^*a*^				
All age groups	76,800 (27,100 to 123,000)	70,600 (21,700 to 120,000)	6,180 (2,770 to 7,900)	8 (2.2 to 21)
5–11 yrs old	10.5 (4.92 to 15)	3.72 (0.984 to 8.39)	6.74 (3.94 to 7.81)	64 (43 to 82)
Vaccinated adults	55,200 (12,500 to 98,800)	50,000 (9,510 to 96,000)	5,210 (2,260 to 6,210)	9.4 (2.6 to 24)
Unvaccinated adults	21,600 (13,800 to 24,400)	20,600 (11,300 to 24,200)	959 (247 to 2,460)	4.4 (1 to 18)
Deaths^*a*^				
All age groups	3,870 (2,540 to 4,570)	3,700 (2,160 to 4,500)	177 (63.4 to 376)	4.6 (1.4 to 15)
5–11 yrs old	3.04 (1.48 to 4.32)	0.61 (0.286 to 0.964)	2.43 (1.2 to 3.36)	80 (78 to 81)
Vaccinated adults	259 (57 to 512)	234 (42.7 to 498)	25.2 (11.5 to 30.6)	9.7 (2.9 to 24)
Unvaccinated adults	3,610 (2,450 to 4,090)	3,460 (2,090 to 4,040)	149 (46.7 to 358)	4.1 (1.1 to 15)
MIS-C cases (0–19 yrs old)	30.7 (14.5 to 44.2)	10.9 (2.89 to 24.7)	19.8 (11.6 to 23)	64 (43 to 82)
Vaccine-related adverse events				
Myocarditis	2.8 (1.3 to 18)	5.8 (2.8 to 36)	−3.0 (−0.48 to −18)^*b*^	−105 (−17 to −650)^*b*^
Anaphylaxis	3.1 (1.4 to 5.1)	6.4 (2.9 to 10)	−3.3 (−1.3 to −8.1)^*b*^	−105 (−42 to −260)^*b*^

a Due to acute COVID-19.

b Negative sign indicates increase in cases with vaccination.

c Numbers in the table represent estimate (95% confidence interval).

Circulation of SARS-CoV-2 strains with different transmissibility could alter model predictions. We therefore ran the model assuming an ℛ_0_ of 2.79, corresponding to the alpha variant (Table S1 in the supplemental material). We also examined the scenario in which a higher proportion of adults was vaccinated (90%) (Table S2).

10.1128/mbio.03789-21.3 TABLE S1 Simulation for Australia (ℛ_0_ = 2.79, 80% of adults vaccinated). Projected differences in cases, hospitalizations, deaths due to COVID-19, multisystem inflammatory syndrome in children (MIS-C), and vaccine adverse events associated with childhood vaccination. Download Table S1, DOCX file, 0.02 MB. Copyright © 2022 Hawkes and Good. 2022 Hawkes and Good. https://creativecommons.org/licenses/by/4.0/ This content is distributed under the terms of the Creative Commons Attribution 4.0 International license.

10.1128/mbio.03789-21.4 TABLE S2 Simulation for Australia (ℛ_0_ = 5.08, 90% of adults vaccinated): Projected differences in cases, hospitalizations, deaths due to COVID-19, multisystem inflammatory syndrome in children (MIS-C), and vaccine adverse events associated with childhood vaccination. Download Table S2, DOCX file, 0.01 MB. Copyright © 2022 Hawkes and Good. 2022 Hawkes and Good. https://creativecommons.org/licenses/by/4.0/ This content is distributed under the terms of the Creative Commons Attribution 4.0 International license.

Next, we performed sensitivity analyses to examine how model outputs varied with (i) the proportion of children vaccinated, and (ii) the intensity of concurrent public health measures. As the proportion of children vaccinated was varied between 0 and 1, an approximately linear relationship was observed in the reduction in cases, hospitalizations, and deaths across all age and vaccine classes (Fig. S1). As the intensity of concurrent public health measures was varied, we found a nonlinear relationship, with a local maximum in the reduction in cases at intermediate values of “social distancing” index (θ) (Fig. S2). A qualitatively similar pattern was observed for hospitalizations and deaths in all age and vaccine classes (Fig. S3).

10.1128/mbio.03789-21.5 FIG S1 Sensitivity analysis of relative reduction in cases, hospitalizations, and deaths, varying the proportion of children vaccinated. The relative (percent) reduction in daily incident cases (A, D, G, and J), hospitalizations (B, E, H, and K), and deaths (C, F, I, and L) for all age groups (A to C), children under 12 (D to F), vaccinated adults (G to I), and unvaccinated adults (J to L) are shown with various childhood vaccination rate. Of note, larger relative effects are seen in children (directly protected through vaccination) than adults (indirectly protected through increased herd immunity) over the range of vaccine uptake. The linear relationship predicts proportional reductions in cases, hospitalizations, and deaths with increasing vaccine uptake, with greatest relative reductions in hospitalizations and deaths in the under 12 age group (E and F) and more modest relative reductions in other age and vaccine classes. Download FIG S1, PDF file, 0.01 MB. Copyright © 2022 Hawkes and Good. 2022 Hawkes and Good. https://creativecommons.org/licenses/by/4.0/ This content is distributed under the terms of the Creative Commons Attribution 4.0 International license.

10.1128/mbio.03789-21.6 FIG S2 Sensitivity analysis. Total cases (over 1 year of epidemic) vary with the intensity of public health prevention measures, as well as childhood vaccination. The solid black line indicates the scenario with no childhood vaccination, the dashed line indicates the scenario with 80% children vaccinated, and the solid red line indicates the relative (percent) reduction. The parameter theta (θ) was used to model changes in the contact rate with public health measures (e.g., social distancing, masking, business and service closures) that will likely continue to be necessary with the highly contagious delta variant (ℛ_0_ = 5.08). In the absence of any control measures (θ = 1), the SIR model predicted that a large fraction of the population (*N_T_*, 25 million) would become infected over the course of the epidemic fourth wave despite high vaccine coverage. The fraction of infected individuals decreased nonlinearly with increasing public health measures. The effect of childhood vaccination was to qualitatively “shift” the curve toward the right. The relative reduction in cases with childhood vaccination yielded a curve with a local maximum around θ of 0.5. This interaction between vaccine coverage and intensity of public health measures can be explained as follows: with strict lockdown, transmission is low, and the epidemic is extinguished and is barely influenced by childhood vaccination; without control measures, transmission of highly infectious delta variant is not prevented by vaccinating children (a small fraction of the total population), but when moderate control measures are in place, reducing the effective reproduction number to near unity (ℛ* ≈ 1), the added benefit of childhood vaccination can “tip” the epidemic toward extinction, yielding a large relative reduction in total cases. Download FIG S2, PDF file, 0.01 MB. Copyright © 2022 Hawkes and Good. 2022 Hawkes and Good. https://creativecommons.org/licenses/by/4.0/ This content is distributed under the terms of the Creative Commons Attribution 4.0 International license.

10.1128/mbio.03789-21.7 FIG S3 Sensitivity analysis of relative reduction in cases, hospitalizations and deaths, varying the intensity of public health measures. By varying theta (θ), a parameter used to model changes in the contact rate with public health measures, from 0 (complete lockdown) to 1 (perfect mixing), we estimated the relative reduction in incident cases (A, D, G, and J), hospitalizations (B, E, H, and K), and deaths (C, F, I, and L) for all age groups (A to C), children under 12 (D to F), vaccinated adults (G to I), and unvaccinated adults (J to L). Model estimates for relative reduction in cases, hospitalizations, and deaths were sensitive to θ, reaching a local maximum around θ of 0.5. This suggests that childhood vaccination and public health measures interact, yielding maximal combined effectiveness at intermediate levels of social distancing. Download FIG S3, PDF file, 0.01 MB. Copyright © 2022 Hawkes and Good. 2022 Hawkes and Good. https://creativecommons.org/licenses/by/4.0/ This content is distributed under the terms of the Creative Commons Attribution 4.0 International license.

## DISCUSSION

In light of the recent introduction of childhood vaccination against SARS-CoV-2, we forecast the effect on child and adult COVID-19 cases, hospitalizations, deaths, complications, and vaccine adverse events in two jurisdictions. Based on our mathematical model (Tables 1 and 2), several observations can be made for children 5 to 11 years of age as follows: (i) high relative (percent) reduction in hospitalizations and deaths; (ii) lower relative reduction in cases because of imperfect vaccine efficacy for prevention of transmission (8); (iii) the absolute reduction in hospitalizations, deaths, and MIS-C was small, given the rarity of these events, even in unvaccinated children (9); and (iv) cases of vaccine-associated myocarditis and anaphylaxis were few (10). For adults, modest herd immunity effects were observed, with relative reduction in hospitalizations and deaths on the order of 8 to 13%. Nonetheless, these correspond to nontrivial reductions in absolute numbers of hospitalizations (∼3,700) and deaths (∼170), mostly among the unvaccinated. Cases of vaccine-associated myocarditis and anaphylaxis were predicted to increase, though case counts remained low.

In addition to projected population health impact, public acceptability will likely play an important role in the implementation of childhood vaccination against SARS-CoV-2. Seasonal influenza provides a benchmark vaccine-preventable respiratory virus against which SARS-CoV-2 public health impacts and vaccination can be compared. There were 349 SARS-CoV-2-related deaths in the first 19 months of the SARS-CoV-2 pandemic in the United States and 116 influenza deaths in the 2019 to 2020 season, respectively (11, 12). Currently, many children in Australia and Alberta do not receive vaccination for seasonal influenza, suggesting that a substantial fraction of parents would be similarly reluctant to vaccinate their children against COVID-19.

Ethical considerations also arise around childhood vaccination. As shown in our model, by vaccinating children, they are benefiting the rest of the community. The argument in favor of this perspective is, however, difficult to prosecute because the people they will be protecting are those older individuals who refuse to be vaccinated or the small percentage of vaccinated people in whom the vaccine is ineffective. An additional ethical question is that of global social justice when administering vaccines to children in high-income settings while vulnerable elderly populations have limited access to vaccine in low-resource settings.

A higher relative impact of childhood vaccination (20 to 30% reduction in overall cases, hospitalizations, and deaths) was observed in models using the SARS-CoV-2 alpha variant with lower transmissibility (ℛ_0_ = 2.79) (Table S1; Text S2) and a higher baseline proportion of vaccinated adults (90%) (Table S2). Moreover, childhood vaccination interacted synergistically with public health measures (θ) to produce a peak relative reduction in cases at intermediate values of θ (Fig. S2). Taken together, these model predictions illustrate that under less intense epidemic conditions (when the effective reproduction rate, ℛ*, is greater than, but close to, 1), modest reductions in the susceptible fraction (as occurs with childhood vaccination) can drive the epidemic toward extinction (ℛ* < 1), resulting in large relative reductions in the disease burden. On the other hand, when ℛ* is high, exponential growth continues with the modest reductions in transmission associated with childhood vaccination; therefore, the relative reduction in disease burden is minor. When ℛ* is less than 1, the epidemic trends toward extinction with or without childhood vaccination; therefore, the relative reduction in disease burden is again minor. The implication for immunization programs is that childhood vaccination likely has the greatest potential for population-wide impact when coupled with other measures (e.g., social distancing, masking, adult vaccination).

10.1128/mbio.03789-21.2 TEXT S2 Model predictions for a different SARS-CoV-2 strain (alpha variant), model predictions for higher vaccination rate among adults, sensitivity analysis of proportion of children vaccinated, and sensitivity analysis of intensity of concurrent public health measures. Download Text S2, DOCX file, 0.01 MB. Copyright © 2022 Hawkes and Good. 2022 Hawkes and Good. https://creativecommons.org/licenses/by/4.0/ This content is distributed under the terms of the Creative Commons Attribution 4.0 International license.

Our modeling study has several limitations. The model was based on a deterministic compartmental susceptible-infected-recovered (SIR) model; thus, stochastic effects were not considered (13). The system of 49 ordinary differential equations (ODEs) was parameterized with vital statistics from Australia or Alberta; therefore, extrapolation to other regions should be done with caution. On the other hand, the age structure and age-specific mortality rates were similar to other high-income, urbanized settings. Age-assortative mixing was incorporated into the model using social contact matrices; however, these were based on prepandemic surveys (14). Other investigators have demonstrated changes in contact rate with evolution of the pandemic and have considered context-specific changes in contact rates (e.g., school closures) (15, 16). We used a composite social distancing index (θ) to capture the combined effects of physical isolation, face masks, and improved hand hygiene on the contact rate (7). This represents a simplification but is justified in the absence of data on the efficacy and uptake of various public health interventions in different age groups. The duration of infection prior to death or recovery was assumed to follow an exponential distribution. Recovered individuals in our model were considered immune (removed permanently from the susceptible pool); we did not incorporate waning immunity in the model. More complex mathematical formulations would be needed to reflect alternative distributions of the duration of infection and immunity. The cocirculation of several virus strains with different transmissibility was not incorporated into the model; however, for the simulation in Australia, we separately considered scenarios with circulating alpha variant (ℛ_0_ = 2.79) and delta variant (ℛ_0_ = 5.03). Vaccine efficacy may differ between strains of SARS-CoV-2, which could alter the model predictions. The impact of other vaccines (e.g., adenovirus-vectored vaccines) was not included in the model; however, mRNA vaccines are the primary vaccine product offered in Australia and Alberta currently.

In summary, our modeling results suggest that childhood vaccination yields modest benefits with minimal risk. Vaccination is predicted to result in substantial relative reductions in child morbidity and mortality, although the absolute reduction is small because these events are rare. Furthermore, the model predicts “altruistic” absolute reductions in adult cases, hospitalizations, and mortality, particularly among the unvaccinated, in whom the risk of these adverse outcomes is high.

## MATERIALS AND METHODS

### Description of the model and model parameters.

**FIG 2:**
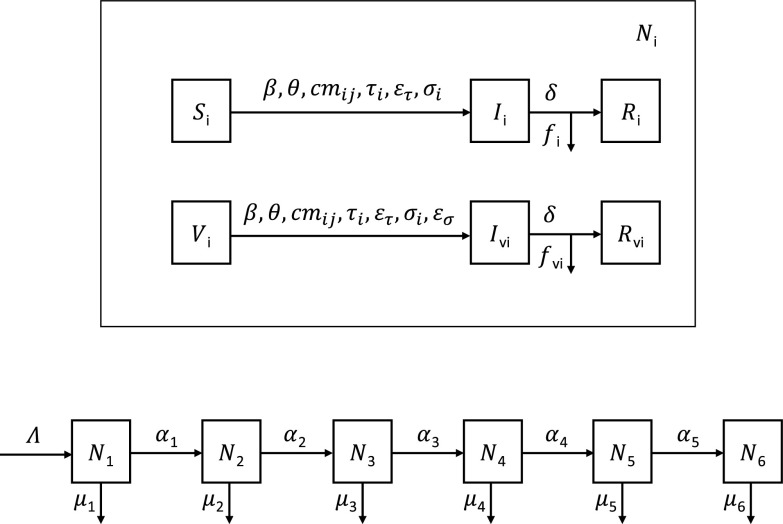
Flowchart for model. The susceptible-infected-recovered (SIR) compartmental model was divided into 7 age classes. This allowed us to incorporate age-specific parameters in the model, including birth rate (Λ), aging between strata (α*_i_*), natural mortality rates (μ*_i_*), social contact matrix (cm*_ij_*), relative infectiousness with SARS-CoV-2 (τ*_i_*), relative susceptibility (σ*_i_*), hospitalization (*h_i_*), and fatality rates (*f_i_*). Estimates for the transmission rate (β) and the duration of infection (1/δ) were taken from previous studies. A parameter theta (θ), reflecting the intensity of public health measures to prevent transmission (e.g., social distancing, mask mandates, service closures) was included to account for reduction in the contact rate from the assumption of perfect mixing. The effect of vaccination was modeled by four parameters, including proportional reduction in infectiousness (ε_τ_), susceptibility (ε_σ_), hospitalization (ε*_h_*), and mortality (ε*_f_*).

We used a deterministic susceptible-infected-recovered (SIR) compartmental model with age structure (seven strata, under 5 years of age, 5 to 11, 12 to 19, 20 to 39, 40 to 59, 60 to 74, and 75 and older) and vaccine with imperfect efficacy (Text S1). The flowchart for this model is shown in Fig. 2. This model accounts for vital dynamics, births (Λ), aging between strata (α*_i_*), and age-specific natural death rate (μ*_i_*). The model includes the following infection parameters: standard incidence ratio (β), contact rate based on published social contact matrices (14) (cm_ij_), age-specific relative infectiousness (τ*_i_*), age-specific relative susceptibility to infection (σ*_i_*), duration of infection (1/δ), and age-specific infection fatality rate (*f_i_*). The effect of vaccination was modeled as relative reduction in infectiousness (ε_τ_), susceptibility to acquiring infection (ε_σ_), probability of hospitalization (ε*_h_*), and mortality (ε*_f_*). To model the changes in the contact rate due to public health measures, we used a social distancing parameter, θ, which varied from zero (complete lockdown) to 1 (complete mixing in the population), as described in a previous modeling study (7). The mathematical properties of this model have been previously discussed (13).

10.1128/mbio.03789-21.1 TEXT S1 Age-structured SIR model parameters, characteristics of SARS-CoV-2 mRNA vaccine that inform model parameters, multisystem inflammatory syndrome in children (MIS-C), and vaccine adverse events. Download Text S1, DOCX file, 0.02 MB. Copyright © 2022 Hawkes and Good. 2022 Hawkes and Good. https://creativecommons.org/licenses/by/4.0/ This content is distributed under the terms of the Creative Commons Attribution 4.0 International license.

Parameter estimates are shown in Table 3. We used realistic estimates based on vital statistics in Australia and Alberta, as well as biological characteristics of SARS-CoV-2. We modeled both the currently dominant and highly infectious delta variant (ℛ_0_ = 5.08) and the historically important alpha variant (ℛ_0_ = 2.79).

**TABLE 3:** Model parameters: values and rationale

Parameter	Estimate	Value^*c*^	Reference
Λ			
Birth rate			
	143 day^−1^ (Alberta)	52,334 births in Alberta (2018)^*a*^	21
	863 day^−1^ (Australia)	315,147 births in Australia (2018)^*a*^	
Population age structure (millions [%])			
Vital statistics			
Australia			22
*N*_1_	1.5 (5.9)	<5	
*N*_2_	2.3 (8.9)	5–11	
*N*_3_	2.5 (9.5)	12–19	
*N*_4_	7.4 (29)	20–39	
*N*_5_	6.4 (25)	40–59	
*N*_6_	3.8 (15)	60–74	
*N*_7_	1.8 (7.1)	≥75	
*N_T_*	25.7 (100)	Total	
Alberta			17
*N*_1_	0.27 (6.6)	<5	
*N*_2_	0.37 (9.1)	5–11	
*N*_3_	0.39 (9.5)	12–19	
*N*_4_	1.2 (30)	20–39	
*N*_5_	1.1 (27)	40–59	
*N*_6_	0.52 (13)	60–74	
*N*_7_	0.21 (5.2)	≥75	
*N_T_*	4.1 (100)	Total	
Aging rate from class *i* to *i* + 1 (per yr)^*a*^			
α_0_	0		13
α_1_	1/5	<5	
α_2_	1/7	5–11	
α_3_	1/8	12–19	
α_4_	1/20	20–39	
α_5_	1/20	40–59	
α_6_	1/15	60–74	
α_7_	0	≥75 (oldest class)	
Natural mortality rate (no. per 1,000 population per yr)			
Vital statistics			
Australia			22
μ_1_	1.1	<5	
μ_2_	0.10	5–11	
μ_3_	0.22	12–19	
μ_4_	0.63	20–39	
μ_5_	2.5	40–59	
μ_6_	9.8	60–74	
μ_7_	54	≥75	
Alberta			17
μ_1_	1.1	<5	
μ_2_	0.10	5–11	
μ_3_	0.22	12–19	
μ_4_	0.88	20–39	
μ_5_	2.8	40–59	
μ_6_	11	60–74	
μ_7_	64	≥75	
Age-specific relative susceptibility to SARS-CoV-2			
σ_1_	0.34	<5	16
σ_2_	0.34	5–11	
σ_3_	0.75	12–19	
σ_4_	1.0 (reference)	20–39	
σ_5_	1.0 (reference)	40–59	
σ_6_	1.26	60–74	
σ_7_	1.47	≥75	
Age-specific relative infectiousness			
τ_1_	0.85	<5	23
τ_2_	0.85	5–11	
τ_3_	0.85	12–19	
τ_4_	1.0 (reference)	20–39	
τ_5_	1.0 (reference)	40–59	
τ_6_	1.0 (reference)	60–74	
τ_7_	1.0 (reference)	≥75	
SARS-CoV-2 hospitalization rate (%)			
*h*_1_	0	<5	9
*h*_2_	0.011	5–11	
*h*_3_	0.041	12–19	
*h*_4_	2.3	20–39	
*h*_5_	6.2	40–59	
*h*_6_	12	60–74	
*h*_7_	16	≥75	
SARS-CoV-2 infection mortality rate (%)			
*f*_1_	0.0016	<5	9
*f*_2_	0.0030	5–11	
*f*_3_	0.0070	12–19	
*f*_4_	0.059	20–39	
*f*_5_	0.38	40–59	
*f*_6_	2.4	60–74	
*f*_7_	6.4	≥75	
Model parameters (%)^*b*^			
β	0.027	Estimated based on ℛ_0_ = 5.08, δ = 1/14 days, and avg contact rate of 13 per day	14, 24
δ	1/14 days^−1^	Mean duration of infection, 14 days to recovery or death	9
θ	0.75	Varied between 0 (complete lockdown) to 1 (perfect mixing) in sensitivity analysis	7
Vaccine efficacy (% [95%CI])			
ε_σ_	67 (37–83)	Reduction in susceptibility	8
ε_τ_	27 (0–62)	Reduction in infectiousness	8
ε*_h_*	86 (82–88)	Prevention of hospitalization	20
ε*_f_*	96.7 (96.0–97.3)	Prevention of fatality	3

a Ages are based on time in each age class.

b β, Standard incidence ratio; δ, rate of recovery from infection; θ, social distancing parameter.

c Data in the “Value” column represent years of age unless otherwise indicated.

A system of 49 ordinary differential equations (ODEs) describes the flow between compartments:

$$\frac{dS_{1}}{\mathrm{dt}}=\Lambda \;-\;\alpha_{1}S_{1}\;-\;{\beta \theta \sigma }_{1}S_{1}\overset{7}{\underset{j=1}{\sum }}\left(\frac{\tau_{j}{\text{cm}}_{\mathrm{ij}}I_{j}}{N_{i}}\;+\;\frac{\tau_{j}(1\;-\;\varepsilon_{\tau }){\text{cm}}_{\mathrm{ij}}I_{\mathrm{vj}}}{N_{i}}\right)\;-\;\mu_{1}S_{1}$$

For *i* = 2,…,7,

$$\frac{dS_{i}}{\mathrm{dt}}=\alpha_{i-1}S_{i-1}\;-\;\alpha_{i}S_{i}\;-\;{\beta \theta \sigma }_{i}S_{i}\overset{7}{\underset{j=1}{\sum }}\left(\frac{\tau_{j}{\text{cm}}_{\mathrm{ij}}I_{j}}{N_{i}}\;+\;\frac{\tau_{j}(1\;-\;\varepsilon_{\tau }){\text{cm}}_{\mathrm{ij}}I_{\mathrm{vj}}}{N_{i}}\right)\;-\;\mu_{i}S_{i}$$

For *i* = 1,2,…,7,

$$\frac{dI_{i}}{\mathrm{dt}}=\alpha_{i-1}I_{i-1}\;-\;\alpha_{i}I_{i}\;+\;{\beta \theta \sigma }_{i}S_{i}\overset{7}{\underset{j=1}{\sum }}\left(\frac{\tau_{j}cm_{\mathrm{ij}}I_{j}}{N_{i}}\;+\;\frac{\tau_{j}(1\;-\;\varepsilon_{\tau }){\text{cm}}_{\mathrm{ij}}I_{\mathrm{vj}}}{N_{i}}\right)\;-\;\delta I_{i}\;-\;\mu_{i}I_{i}$$

$$\frac{dR_{i}}{\mathrm{dt}}=\alpha_{i-1}R_{i-1}\;-\;\alpha_{i}R_{i}\;+\;\delta \left(1\;-\;f_{i}\right)I_{i}\;-\;\mu_{i}R_{i}$$

$$\frac{dV_{i}}{\mathrm{dt}}=\alpha_{i-1}V_{i-1}\;-\;\alpha_{i}V_{i}\;-\;{\beta \theta \sigma }_{i}(1\;-\;\varepsilon_{\sigma })V_{i}\overset{7}{\underset{j=1}{\sum }}\left(\frac{\tau_{j}cm_{\mathrm{ij}}I_{j}}{N_{i}}\;+\;\frac{\tau_{j}(1\;-\;\varepsilon_{\tau })cm_{\mathrm{ij}}I_{\mathrm{vj}}}{N_{i}}\right)\;-\;\mu_{i}V_{i}$$

$$\frac{dI_{\mathrm{vi}}}{\mathrm{dt}}=\alpha_{i-1}I_{\mathrm{vi}-1}\;-\;\alpha_{i}I_{\mathrm{vi}}\;+\;{\beta \theta \sigma }_{i}(1\;-\;\varepsilon_{\sigma })V_{i}\overset{7}{\underset{j=1}{\sum }}\left(\frac{\tau_{j}cm_{\mathrm{ij}}I_{j}}{N_{i}}\;+\;\frac{\tau_{j}(1\;-\;\varepsilon_{\tau })cm_{\mathrm{ij}}I_{\mathrm{vj}}}{N_{i}}\right)\;-\;\delta I_{\mathrm{vi}}\;-\;\mu_{i}I_{\mathrm{vi}}$$

$$\frac{dR_{\mathrm{vi}}}{\mathrm{dt}}=\alpha_{i-1}R_{\mathrm{vi}-1}\;-\;\alpha_{i}R_{\mathrm{vi}}\;+\;\delta \left(1\;-\;f_{\mathrm{vi}}\right)I_{i}\;-\;\mu_{i}R_{\mathrm{vi}}$$

$$\frac{dD_{i}}{\mathrm{dt}}=\delta f_{i}I_{i}\;+\;\delta f_{\mathrm{vi}}I_{\mathrm{vi}}$$

$$N_{i}=S_{i}\;+\;I_{i}\;+\;R_{i}\;+\;V_{i}\;+\;I_{\mathrm{vi}}\;+\;R_{\mathrm{vi}}$$

$$h_{\mathrm{vi}}=h_{i}(1\;-\;\varepsilon_{h})$$

$$f_{\mathrm{vi}}=f_{i}(1\;-\;\varepsilon_{f})$$

### Initial conditions and time horizon.

To model the course of a future “wave” of SARS-CoV-2, we began with initial conditions which included the total population of Australia or Alberta, divided into age classes, and further subdivided into vaccinated and unvaccinated compartments (17). The proportion of actively infected individuals was calculated based on the number of known active cases in Australia or Alberta in August 2021, proportionally divided among the age classes. We assumed that all these cases would be isolated and, therefore, would not contribute to the infectious pool. We further assumed that a 5-fold higher number of undiagnosed cases would be present in the community. This assumption is based on a previous study, which estimated that the number of infections in the United States was 3 to 20 times higher than the number of confirmed cases (4). The proportion of recovered individuals was based on seroprevalence surveys (18, 19). In hypothetical scenarios for Australia, 80% or 90% of adults were presumed to be vaccinated at baseline. In the Alberta scenario, the proportion of vaccinated individuals in each age stratum in October 2021 was used as the baseline. The model was run with no childhood vaccination and with 80% childhood vaccination. The model was run for a period of 365 days. In the absence of an analytical solution to the system of ODEs, we used numerical simulations (package deSolver) in the R statistical environment (R version 3.6.2).

### Confidence intervals for model outputs.

To account for uncertainties in the vaccine efficacy, we used a multiway sensitivity analysis, varying the four key parameters (efficacy to prevent transmission, susceptibility, hospitalization, and death) over their 95% confidence interval, based on published studies (3, 8, 20). We assumed that each proportion followed a beta distribution. We randomly sampled from the distribution of each parameter, used these as inputs for the model, and ran the SIR model 1,000 times. Using the distribution of model outputs that was generated, the 95% confidence interval for each output was defined by the 2.5th percentile and the 97.5th percentile.

### Variation of model estimates with vaccine uptake and concurrent public health measures.

The proportion of vaccinated children and public health measures may vary widely over time and geography. Therefore, we varied these key parameters from 0 to 1 (over the entire possible range) and plotted the resulting model outputs. Graphical methods were used to examine the dependence of model outputs on key parameters.

## References

[1] Hothorn T, Bopp M, Günthard H, Keiser O, Roelens M, Weibull CE, Crowther M. 2021. Assessing relative COVID-19 mortality: a Swiss population-based study. BMJ Open 11:e042387. doi:10.1136/bmjopen-2020-042387. PMC7941676 34006026

[2] McCormick DW, Richardson LC, Young PR, Viens LJ, Gould CV, Kimball A, Pindyck T, Rosenblum HG, Siegel DA, Vu QM, Komatsu K, Venkat H, Openshaw JJ, Kawasaki B, Siniscalchi AJ, Gumke M, Leapley A, Tobin-D’Angelo M, Kauerauf J, Reid H, White K, Ahmed FS, Richardson G, Hand J, Kirkey K, Larson L, Byers P, Garcia A, Ojo M, Zamcheck A, Lash MK, Lee EH, Reilly KH, Wilson E, de Fijter S, Naqvi OH, Harduar-Morano L, Burch A-K, Lewis A, Kolsin J, Pont SJ, Barbeau B, Bixler D, Reagan-Steiner S, Koumans EH. 2021. Deaths in children and adolescents associated with COVID-19 and MIS-C in the United States. Pediatrics 148:e2021052273. doi:10.1542/peds.2021-052273. 34385349 PMC9837742

[3] Haas EJ, Angulo FJ, McLaughlin JM, Anis E, Singer SR, Khan F, Brooks N, Smaja M, Mircus G, Pan K, Southern J, Swerdlow DL, Jodar L, Levy Y, Alroy-Preis S. 2021. Impact and effectiveness of mRNA BNT162b2 vaccine against SARS-CoV-2 infections and COVID-19 cases, hospitalisations, and deaths following a nationwide vaccination campaign in Israel: an observational study using national surveillance data. Lancet 397:1819–1829. doi:10.1016/S0140-6736(21)00947-8. 33964222 PMC8099315

[4] Wu SL, Mertens AN, Crider YS, Nguyen A, Pokpongkiat NN, Djajadi S, Seth A, Hsiang MS, Colford JM, Reingold A, Arnold BF, Hubbard A, Benjamin-Chung J. 2020. Substantial underestimation of SARS-CoV-2 infection in the United States. Nat Commun 11:4507. doi:10.1038/s41467-020-18272-4. 32908126 PMC7481226

[5] Liu Y, Rocklov J. 2021. The reproductive number of the delta variant of SARS-CoV-2 is far higher compared to the ancestral SARS-CoV-2 virus. J Travel Med 28:taab124. doi:10.1093/jtm/taab124. 34369565 PMC8436367

[6] Harris RJ, Hall JA, Zaidi A, Andrews NJ, Dunbar JK, Dabrera G. 2021. Effect of vaccination on household transmission of SARS-CoV-2 in England. N Engl J Med 385:759–760. doi:10.1056/NEJMc2107717. 34161702 PMC8262621

[7] Good MF, Hawkes MT. 2020. The interaction of natural and vaccine-induced immunity with social distancing predicts the evolution of the COVID-19 pandemic. mBio 11:e02617-20. doi:10.1128/mBio.02617-20. 33097654 PMC7587444

[8] Ng OT, Koh V, Chiew CJ, Marimuthu K, Thevasagayam NM, Mak TM, Chua JK, Ong SSH, Lim YK, Ferdous Z, Johari AKB, Chen MI-C, Maurer-Stroh S, Cui L, Lin RTP, Tan KB, Cook AR, Leo PY-S, Lee PVJ. 2021. Impact of delta variant and vaccination on SARS-CoV-2 secondary attack rate among household close contacts. Lancet Reg Health West Pac 17:100299. doi:10.1016/j.lanwpc.2021.100299. 34746899 PMC8560026

[9] Verity R, Okell LC, Dorigatti I, Winskill P, Whittaker C, Imai N, Cuomo-Dannenburg G, Thompson H, Walker PGT, Fu H, Dighe A, Griffin JT, Baguelin M, Bhatia S, Boonyasiri A, Cori A, Cucunubá Z, FitzJohn R, Gaythorpe K, Green W, Hamlet A, Hinsley W, Laydon D, Nedjati-Gilani G, Riley S, van Elsland S, Volz E, Wang H, Wang Y, Xi X, Donnelly CA, Ghani AC, Ferguson NM. 2020. Estimates of the severity of coronavirus disease 2019: a model-based analysis. Lancet Infect Dis 20:669–677. doi:10.1016/S1473-3099(20)30243-7. 32240634 PMC7158570

[10] Shay DK, Shimabukuro TT, DeStefano F. 2021. Myocarditis occurring after immunization with mRNA-based COVID-19 vaccines. JAMA Cardiol 6:1115. doi:10.1001/jamacardio.2021.2821. 34185047

[11] Garten R, Blanton L, Elal AIA, Alabi N, Barnes J, Biggerstaff M, Brammer L, Budd AP, Burns E, Cummings CN, Davis T, Garg S, Gubareva L, Jang Y, Kniss K, Kramer N, Lindstrom S, Mustaquim D, O'Halloran A, Sessions W, Taylor C, Xu X, Dugan VG, Fry AM, Wentworth DE, Katz J, Jernigan D. 2018. Update: influenza activity in the United States during the 2017–18 season and composition of the 2018–19 influenza vaccine. MMWR Morb Mortal Wkly Rep 67:634–642. doi:10.15585/mmwr.mm6722a4. 29879098 PMC5991814

[12] Xu X, Blanton L, Elal AIA, Alabi N, Barnes J, Biggerstaff M, Brammer L, Budd AP, Burns E, Cummings CN, Garg S, Kondor R, Gubareva L, Kniss K, Nyanseor S, O'Halloran A, Rolfes M, Sessions W, Dugan VG, Fry AM, Wentworth DE, Stevens J, Jernigan D. 2019. Update: influenza activity in the United States during the 2018–19 season and composition of the 2019–20 influenza vaccine. MMWR Morb Mortal Wkly Rep 68:544–551. doi:10.15585/mmwr.mm6824a3. 31220057 PMC6586370

[13] Magpantay FMG. 2017. Vaccine impact in homogeneous and age-structured models. J Math Biol 75:1591–1617. doi:10.1007/s00285-017-1126-5. 28417166 PMC5643245

[14] Prem K, Cook AR, Jit M. 2017. Projecting social contact matrices in 152 countries using contact surveys and demographic data. PLoS Comput Biol 13:e1005697. doi:10.1371/journal.pcbi.1005697. 28898249 PMC5609774

[15] Zhang J, Klepac P, Read JM, Rosello A, Wang X, Lai S, Li M, Song Y, Wei Q, Jiang H, Yang J, Lynn H, Flasche S, Jit M, Yu H. 2019. Patterns of human social contact and contact with animals in Shanghai, China. Sci Rep 9:15141. doi:10.1038/s41598-019-51609-8. 31641189 PMC6805924

[16] Zhang J, Litvinova M, Liang Y, Wang Y, Wang W, Zhao S, Wu Q, Merler S, Viboud C, Vespignani A, Ajelli M, Yu H. 2020. Changes in contact patterns shape the dynamics of the COVID-19 outbreak in China. Science 368:1481–1486. doi:10.1126/science.abb8001. 32350060 PMC7199529

[17] Statistics Canada. 2016. Census profile, 2016 census. https://www12.statcan.gc.ca/census-recensement/2016. Accessed 20 August 2021.

[18] Gidding HF, Machalek DA, Hendry AJ, Quinn HE, Vette K, Beard FH, Shilling HS, Hirani R, Gosbell IB, Irving DO, Hueston L, Downes M, Carlin JB, O'Sullivan MV, Dwyer DE, Kaldor JM, Macartney K. 2021. Seroprevalence of SARS-CoV-2-specific antibodies in Sydney after the first epidemic wave of 2020. Med J Aust 214:179–185. doi:10.5694/mja2.50940. 33538019 PMC8014239

[19] Statistics Canada. 2021. Canadian COVID-19 antibody and health survey. Provincial or regional SARS-CoV-2 antibody seroprevalence estimates, by antibody seroprevalence type. https://www150.statcan.gc.ca/n1/daily-quotidien/210706/t003a-eng.htm. Accessed 20 August 2021.

[20] Tenforde MW, Self WH, Naioti EA, Ginde AA, Douin DJ, Olson SM, Talbot HK, Casey JD, Mohr NM, Zepeski A, Gaglani M, McNeal T, Ghamande S, Shapiro NI, Gibbs KW, Files DC, Hager DN, Shehu A, Prekker ME, Erickson HL, Gong MN, Mohamed A, Henning DJ, Steingrub JS, Peltan ID, Brown SM, Martin ET, Monto AS, Khan A, Hough CL, Busse LW, Ten Lohuis CC, Duggal A, Wilson JG, Gordon AJ, Qadir N, Chang SY, Mallow C, Rivas C, Babcock HM, Kwon JH, Exline MC, Halasa N, Chappell JD, Lauring AS, Grijalva CG, Rice TW, Jones ID, Stubblefield WB, Baughman A, et al. 2021. Sustained effectiveness of Pfizer-BioNTech and Moderna vaccines against COVID-19 associated hospitalizations among adults - United States, March-July 2021. MMWR Morb Mortal Wkly Rep 70:1156–1162. doi:10.15585/mmwr.mm7034e2. 34437524 PMC8389395

[21] Alberta Government. 2020. Vital statistics (births and deaths) - Alberta, census divisions and economic regions. https://open.alberta.ca/opendata/vital-statistics-births-and-deaths-alberta-census-divisions-economic-regions. Accessed 27 July 2020.

[22] Australian Bureau of Statistics. Deaths, year of registration, age at death, age-specific death rates, sex, states, territories and Australia. https://www.abs.gov.au/statistics/people/population/deaths-australia/latest-release. Accessed 20 August 2021.

[23] Dattner I, Goldberg Y, Katriel G, Yaari R, Gal N, Miron Y, Ziv A, Sheffer R, Hamo Y, Huppert A. 2021. The role of children in the spread of COVID-19: using household data from Bnei Brak, Israel, to estimate the relative susceptibility and infectivity of children. PLoS Comput Biol 17:e1008559. doi:10.1371/journal.pcbi.1008559. 33571188 PMC7877572

[24] Zhao S, Lin Q, Ran J, Musa SS, Yang G, Wang W, Lou Y, Gao D, Yang L, He D, Wang MH. 2020. Preliminary estimation of the basic reproduction number of novel coronavirus (2019-nCoV) in China, from 2019 to 2020: a data-driven analysis in the early phase of the outbreak. Int J Infect Dis 92:214–217. doi:10.1016/j.ijid.2020.01.050. 32007643 PMC7110798

