# Supplementary material for: Vaccinating Children against COVID-19: Commentary and Mathematical Modeling
Source: mBio. 2022 Jan 18;13(1):e03789-21. doi: 10.1128/mbio.03789-21 (PMC8764932; doi:10.1128/mbio.03789-21)
Supplement: TABLE S1 [file mbio.03789-21-st001.docx]

# Table S1. Simulation for Australia (ℛ_0_ = 2.79, 80% of adults vaccinated): Projected differences in cases, hospitalizations, deaths due to COVID-19, multisystem inflammatory syndrome in children (MIS-C) and vaccine adverse events associated with childhood vaccination

|  | **No childhood vaccination** | **Childhood vaccination**  **(80% coverage)** | **Absolute reduction** | **Relative reduction (%)** |
| --- | --- | --- | --- | --- |
| **Cases of COVID-19 (×1000)** |  |  |  |  |
| All age groups | 430 (88.1-7830) | 305 (72.5-6780) | 125 (15.6-1220) | 29 (13-37) |
| Under 12 years old | 15.5 (5.51-240) | 4.46 (1.26-115) | 11 (4.16-123) | 71 (52-78) |
| Vaccinated adults | 233 (28.4-5280) | 170 (24.6-4660) | 63 (3.74-738) | 27 (12-35) |
| Unvaccinated adults | 178 (50.4-2210) | 127 (43.6-1950) | 50.2 (6.72-382) | 28 (12-36) |
| **Hospitalizations**^1^ |  |  |  |  |
| All age groups | 17800 (3590-337000) | 13000 (3130-297000) | 4750 (456-48300) | 27 (11-35) |
| Under 12 years old | 1.66 (0.592-25.8) | 0.479 (0.136-12.3) | 1.18 (0.447-13.3) | 71 (52-78) |
| Vaccinated adults | 10200 (1260-238000) | 7450 (1100-208000) | 2730 (162-33600) | 27 (11-35) |
| Unvaccinated adults | 7610 (2220-98700) | 5590 (1950-88900) | 2020 (274-15100) | 27 (11-35) |
| **Deaths^1^** |  |  |  |  |
| All age groups | 1630 (786-16600) | 1320 (745-14700) | 307 (41.7-2420) | 19 (5.2-30) |
| Under 12 years old | 0.569 (0.274-7.24) | 0.175 (0.135-1.51) | 0.393 (0.138-5.66) | 69 (50-82) |
| Vaccinated adults | 50 (6.31-1180) | 36.9 (5.49-1050) | 13 (0.797-174) | 26 (11-34) |
| Unvaccinated adults | 1580 (779-15500) | 1280 (738-13900) | 294 (40.7-2250) | 19 (5.1-30) |
| **MIS-C cases (0-19 years old)** | 4.89 (1.74-75.9) | 1.41 (0.399-36.3) | 3.48 (1.31-39) | 71 (52-78) |
| **Vaccine-related adverse events** |  |  |  |  |
| Myocarditis | 20 (9.0-120) | 38 (18-240) | -18 (-3.0 to -110)^2^ | -93 (-15 to -570)^2^ |
| Anaphylaxis | 22 (9.8-35) | 42 (19-68) | -20 (-8.1 to -50)^2^ | -92 (-37 to -230)^2^ |

^1^Due to acute COVID-19

^2^Negative sign indicates increase in cases with vaccination
