# Supplementary material for: Vaccinating Children against COVID-19: Commentary and Mathematical Modeling
Source: mBio. 2022 Jan 18;13(1):e03789-21. doi: 10.1128/mbio.03789-21 (PMC8764932; doi:10.1128/mbio.03789-21)
Supplement: TABLE S2 [file mbio.03789-21-st002.docx]

# Table S2. Simulation for Australia (ℛ_0_ = 5.08, 90% of adults vaccinated): Projected differences in cases, hospitalizations, deaths due to COVID-19, multisystem inflammatory syndrome in children (MIS-C) and vaccine adverse events associated with childhood vaccination

|  | **No childhood vaccination** | **Childhood vaccination**  **(80% coverage)** | **Absolute reduction** | **Relative reduction (%)** |
| --- | --- | --- | --- | --- |
| **Cases of COVID-19 (×1000)** |  |  |  |  |
| All age groups | 9510 (321-17200) | 7010 (150-16200) | 2510 (166-3140) | 26 (5.8-71) |
| Under 12 years old | 589 (29.6-971) | 154 (3.43-501) | 435 (26.5-535) | 74 (46-93) |
| Vaccinated adults | 7130 (167-14100) | 5440 (78.9-13500) | 1690 (76.7-2240) | 24 (3-70) |
| Unvaccinated adults | 1660 (109-1990) | 1320 (55.8-1890) | 345 (53.3-653) | 21 (5.1-69) |
| **Hospitalizations**^1^ |  |  |  |  |
| All age groups | 398000 (12200-785000) | 305000 (6350-757000) | 93300 (5720-122000) | 23 (3.8-69) |
| Under 12 years old | 63.3 (3.18-104) | 16.6 (0.369-53.9) | 46.7 (2.85-57.4) | 74 (46-93) |
| Vaccinated adults | 323000 (7240-694000) | 243000 (3480-659000) | 79800 (3480-101000) | 25 (4-69) |
| Unvaccinated adults | 74800 (4570-101000) | 61400 (2470-98100) | 13400 (1780-26300) | 18 (2.6-67) |
| **Deaths^1^** |  |  |  |  |
| All age groups | 13900 (1150-20900) | 11100 (834-20200) | 2740 (314-4400) | 20 (3.6-60) |
| Under 12 years old | 17.7 (0.986-29.3) | 2.45 (0.174-5.78) | 15.2 (0.813-23.5) | 86 (80-93) |
| Vaccinated adults | 1620 (34.6-3700) | 1210 (17-3550) | 409 (16.7-528) | 25 (4.5-68) |
| Unvaccinated adults | 12200 (1120-17300) | 9930 (818-16700) | 2320 (302-4010) | 19 (3.3-59) |
| **MIS-C cases (0-19 years old)** | 186 (9.35-307) | 48.8 (1.09-158) | 137 (8.38-169) | 74 (46-93) |
| **Vaccine-related adverse events** |  |  |  |  |
| Myocarditis | 20 (9.0-120) | 38 (18-240) | -18 (-3.0 to -110)^2^ | -93 (-15 to -570)^2^ |
| Anaphylaxis | 22 (9.8-35) | 42 (19-68) | -20 (-8.1 to -50)^2^ | -92 (-37 to -230)^2^ |

^1^Due to acute COVID-19

^2^Negative sign indicates increase in cases with vaccination
