# Supplementary figures and images for: Vaccinating Children against COVID-19: Commentary and Mathematical Modeling
Source: mBio. 2022 Jan 18;13(1):e03789-21. doi: 10.1128/mbio.03789-21 (PMC8764932; doi:10.1128/mbio.03789-21)

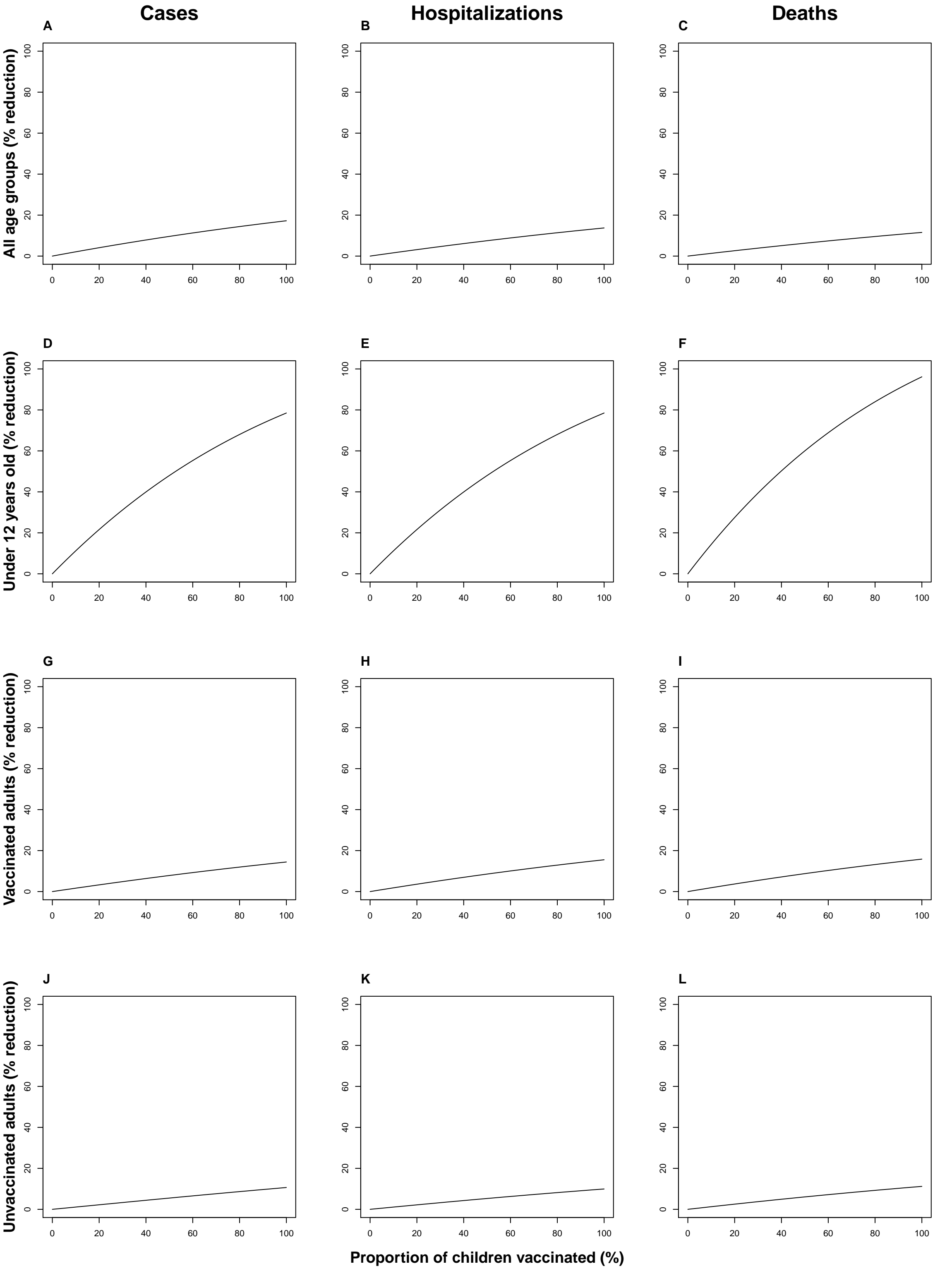

Supplement: FIG S1 [file mbio.03789-21-sf001.pdf]

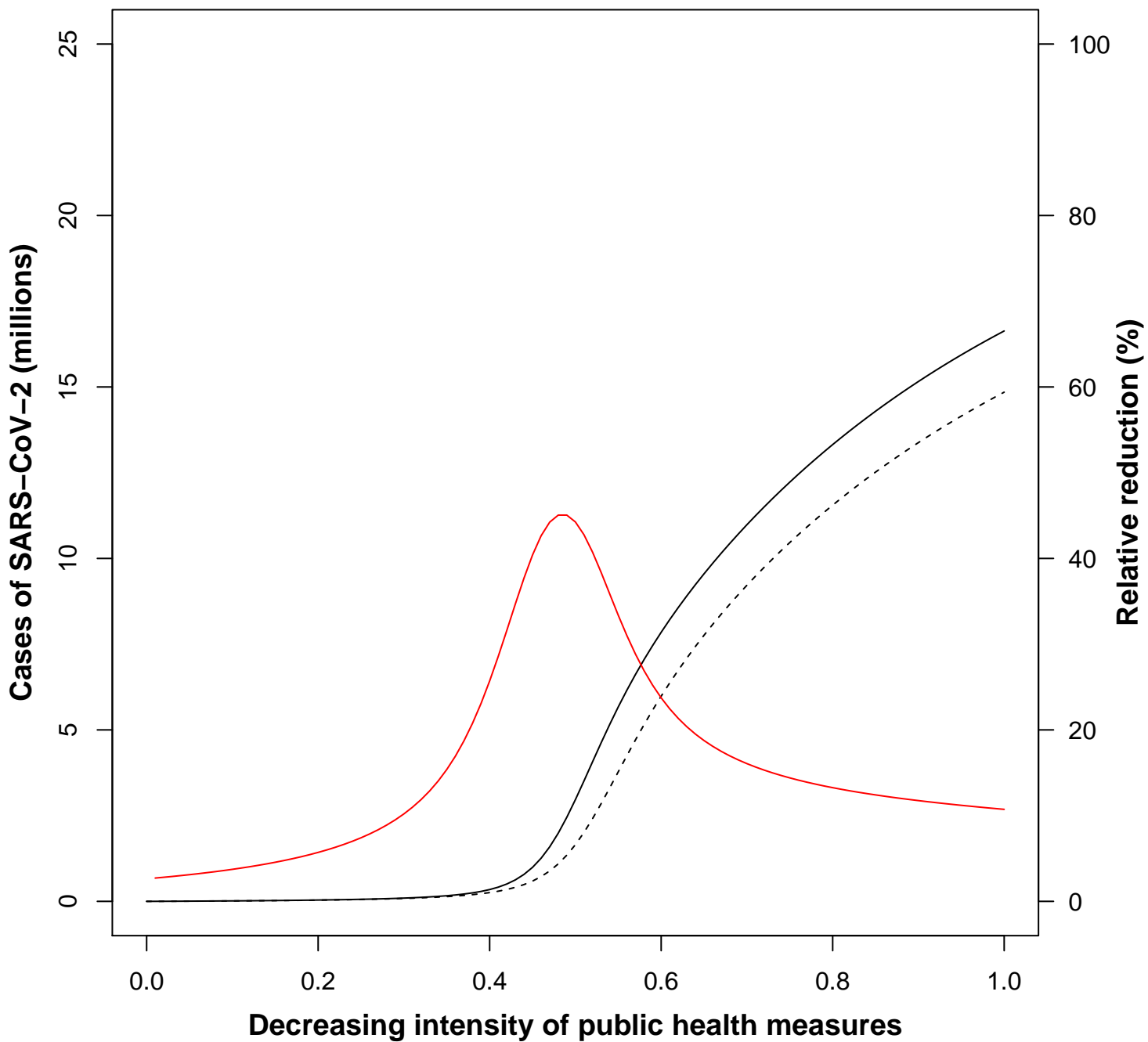

Supplement: FIG S2 [file mbio.03789-21-sf002.pdf]

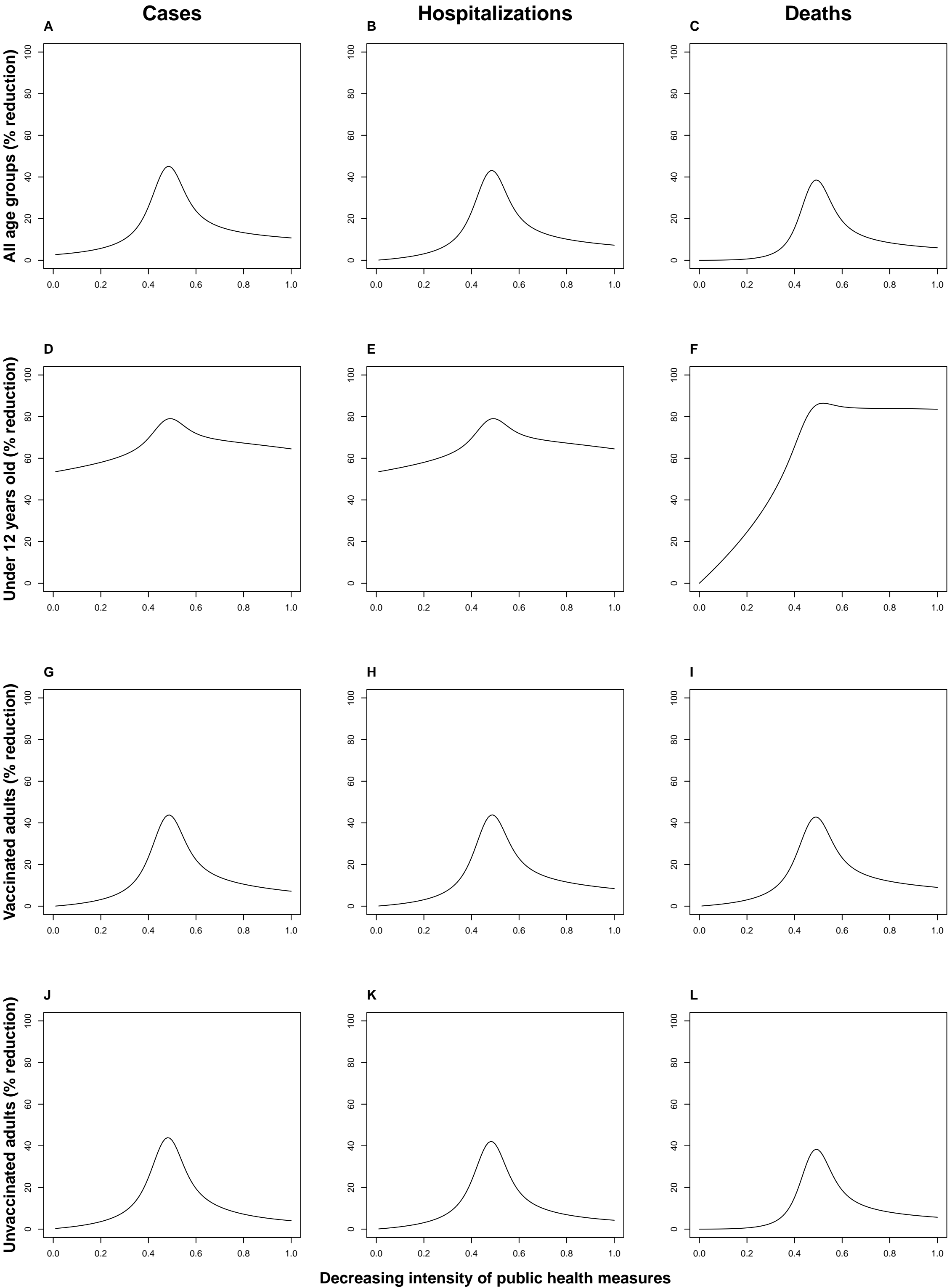

Supplement: FIG S3 [file mbio.03789-21-sf003.pdf]
