# Supplementary material for: Vaccinating Children against COVID-19: Commentary and Mathematical Modeling
Source: mBio. 2022 Jan 18;13(1):e03789-21. doi: 10.1128/mbio.03789-21 (PMC8764932; doi:10.1128/mbio.03789-21)
Supplement: TEXT S2 [file mbio.03789-21-t0002.docx]

# Supplemental Results

## Model predictions for a different SARS-CoV-2 strain (alpha variant)

Because of variations in transmissibility, co-circulation or change in the dominant circulating strain could affect model predictions. In addition to the delta variant (ℛ_0_ = 5.08) presented in the main manuscript, we modelled the course of the epidemic with the alpha variant (ℛ_0_ = 2.79). The results over one year are shown in Table S1.

## Model predictions for higher vaccination rate among adults

As of 28 Nov, 2021, 86.7% of Australians over the age of 16 years had received two doses of vaccine.^21^ We therefore considered a separate case in which the rate of adult vaccination was 90% (versus 80% in Table 2, main manuscript). The results over one year are shown in Table S2.

## Sensitivity analysis: proportion of children vaccinated

Next, we examined the effect of varying the proportion of children vaccinated on the model outputs. A linear relationship was observed in absolute reduction (data not shown) and relative reduction in cases, hospitalizations, and deaths in all age and vaccine classes (Figure S1). Conveniently, this linear relationship would allow the predicted effect of less complete vaccine coverage to be calculated in a straightforward manner.

## Sensitivity analysis: intensity of concurrent public health measures

In a sensitivity analysis of the effect of childhood vaccination, varying the intensity of public health measures ($\theta$ in the SIR model), we found a non-linear relationship with a local maximum in relative reduction of SARS-CoV-2 cases at intermediate values of $\theta$ (Figure S2). A qualitatively similar pattern was observed for the number of hospitalizations and the number of deaths (data not shown). A similar pattern was seen for other model outcomes (hospitalizations and deaths) in all age and vaccine classes (Figure S3). This analysis suggests an interaction between childhood vaccination and public health measures, with highest effect of childhood vaccination when some public health measures remain in place.
