# Supplementary material for: Vaccinating Children against COVID-19: Commentary and Mathematical Modeling
Source: mBio. 2022 Jan 18;13(1):e03789-21. doi: 10.1128/mbio.03789-21 (PMC8764932; doi:10.1128/mbio.03789-21)
Supplement: TEXT S1 [file mbio.03789-21-t0001.docx]

# Supplemental background information

## Age-structured SIR model parameters

Age-specific contact rates were quantified using published social contact matrices, specific for each geographic setting.^1^ In the referenced studies, these contact matrices were determined by survey methodology (number of people in each age stratum who engaged in a two-way conversation involving three or more words in the physical presence of the other person or direct physical contact) and statistical projections based on demographic information about the country.^1^ We used a series of transformations to account for the desired age group divisions and to satisfy the condition of reciprocity between age groups.^2^

Age-specific susceptibility to SARS-CoV-2 was incorporated into the model, based on the observation that, compared to a baseline risk in adults 15 to 64 years of age, children 0 to 14 years of age were less susceptible (odds ratio 0.34), and elderly individuals more than 65 years of age were more susceptible (odds ratio 1.47).^3^ Age variation in infectiousness was also included in the model, based on a study from Israel indicating that the relative infectiousness for children and youth younger than 20 years of age was 0.85, compared to adults.^4^ Other studies support the lower infectiousness of children.^5-7^

## Characteristics of SARS-CoV-2 mRNA vaccine that inform model parameters

We reviewed the published literature to determine the best available data for vaccine efficacy. There are several possible clinically relevant outcomes of interest, including prevention of incident cases (through reduction in infectiousness and/or susceptibility to acquiring infection), hospitalizations, and deaths. We focused on the leading BNT162b2 mRNA vaccine (Comirnaty, Pfizer) since this vaccine has been most widely administered in North America and Australia and has the most detailed efficacy data. Where possible, we included data specific to the SARS-CoV-2 delta variant, since this is currently the dominant strain globally.

Vaccination has been shown to decrease both peak viral load and the duration of infection when breakthrough infection occurs.^8,9^ The efficacy of the vaccine in reducing infectiousness was directly studied among vaccinated and unvaccinated health workers in the UK.^10^ Household contacts had a reduced odds of acquiring SARS-CoV-2 from cases who had been vaccinated with the BNT162b2 mRNA vaccine compared to unvaccinated cases, although these data were collected during a phase of the epidemic when the alpha variant was circulating.^10^ In a retrospective cohort from Singapore, investigators were able to examine the vaccine efficacy against transmission of the delta variant.^11^ In addition, they were able to disentangle the effect of vaccination on susceptibility and infectiousness: the relative odds of household transmission was 0.33 if the contact was vaccinated compared to an unvaccinated contact (reduced susceptibility) and 0.73 if the index had been previously vaccinated compared to an unvaccinated index case (reduced infectiousness).^11^

SARS-CoV-2-related hospitalization is an important public health outcome, since epidemic waves threaten to overwhelm healthcare services. The vaccine efficacy in preventing hospitalization from SARS-CoV-2 was 86% (95%CI 82%–88%) in a recent analysis by the US CDC.^12^

With respect to mortality benefit of the vaccine, based on national surveillance data following a nationwide vaccination campaign in Israel, BNT162b2 mRNA vaccine was 96.7% effective at preventing death from SARS-CoV-2 (95% CI 96.0-97.3%).^13^

## Multisystem inflammatory syndrome in children (MIS-C)

We included MIS-C as a distinct, serious, and occasionally fatal complication of COVID-19 in children.^14^ Incidence of MIS-C was estimated to be 316 persons per million SARS-CoV-2 infections in persons younger than 21 years.^15^ Deaths due to MIS-C are uncommon, and accounted for only 14% of decedents in a study of deaths due to COVID-19 among children and adolescents in the US.^16^

## Vaccine adverse events

With respect to adverse reaction associated with COVID-19 mRNA vaccines, two distinct self-limited cardiac syndromes, myocarditis and pericarditis, have been reported.^17^ These adverse events have not been studied in children under 12 years of age. Therefore, we extrapolated the rate of these adverse events from available data in older children and young adults. Myocarditis occurred soon after immunization, in younger patients, mostly after the second vaccination.^17^ Pericarditis affected older patients later, after either the first or second dose.^17^ Because we were interested in the effects of childhood vaccination, we modelled the increase in myocarditis cases, since this was the childhood-specific severe adverse event.^18^ The incidence of myocarditis has been estimated at 10 to 63 per million doses with the highest incidence among males aged 12−17 years.^17,18^ No deaths have been reported for young adults who developed myocarditis after being given the mRNA vaccines, despite 1,226 reports of myocarditis reported in the US. However, a single death due to myocarditis has recently been reported from New Zealand.^19^ Because of the apparent rarity of this event, deaths from vaccine-associated myocarditis were not counted in our model.

The US CDC detected 21 cases of anaphylaxis following 1.9 million doses of the Pfizer-BioNTech mRNA SARS-CoV-2 vaccine (11.1 cases per million doses).^20^ Most (71%) cases occurred within 15 minutes of vaccination and there were no fatalities.^20^ We included the predicted number of cases of anaphylaxis in our model; however, deaths from anaphylaxis were not counted.

# References

1. Prem K, Cook AR, Jit M. Projecting social contact matrices in 152 countries using contact surveys and demographic data. *PLoS Comput Biol* 2017; **13**(9): e1005697.

2. McCarthy Z, Xiao Y, Scarabel F, et al. Quantifying the shift in social contact patterns in response to non-pharmaceutical interventions. *J Math Ind* 2020; **10**(1): 28.

3. Zhang J, Litvinova M, Liang Y, et al. Changes in contact patterns shape the dynamics of the COVID-19 outbreak in China. *Science* 2020; **368**(6498): 1481-6.

4. Dattner I, Goldberg Y, Katriel G, et al. The role of children in the spread of COVID-19: Using household data from Bnei Brak, Israel, to estimate the relative susceptibility and infectivity of children. *PLoS Comput Biol* 2021; **17**(2): e1008559.

5. Goldstein E, Lipsitch M, Cevik M. On the Effect of Age on the Transmission of SARS-CoV-2 in Households, Schools, and the Community. *J Infect Dis* 2021; **223**(3): 362-9.

6. Laxminarayan R, Wahl B, Dudala SR, et al. Epidemiology and transmission dynamics of COVID-19 in two Indian states. *Science* 2020; **370**(6517): 691-7.

7. Hu S, Wang W, Wang Y, et al. Infectivity, susceptibility, and risk factors associated with SARS-CoV-2 transmission under intensive contact tracing in Hunan, China. *Nat Commun* 2021; **12**(1): 1533.

8. Levine-Tiefenbrun M, Yelin I, Katz R, et al. Initial report of decreased SARS-CoV-2 viral load after inoculation with the BNT162b2 vaccine. *Nat Med* 2021; **27**(5): 790-2.

9. Damiani V, Mandatori D, De Fabritiis S, et al. Severe acute respiratory coronavirus virus 2 (SARS-CoV-2) infection in asymptomatic vaccinated healthcare workers. *Infect Control Hosp Epidemiol* 2021: 1-2.

10. Harris RJ, Hall JA, Zaidi A, Andrews NJ, Dunbar JK, Dabrera G. Effect of Vaccination on Household Transmission of SARS-CoV-2 in England. *N Engl J Med* 2021; **385**(8): 759-60.

11. Ng OT, Koh V, Chiew CJ, et al. Impact of Delta Variant and Vaccination on SARS-CoV-2 Secondary Attack Rate Among Household Close Contacts. *Lancet Reg Health West Pac* 2021; **17**: 100299.

12. Tenforde MW, Self WH, Naioti EA, et al. Sustained Effectiveness of Pfizer-BioNTech and Moderna Vaccines Against COVID-19 Associated Hospitalizations Among Adults - United States, March-July 2021. *MMWR Morb Mortal Wkly Rep* 2021; **70**(34): 1156-62.

13. Haas EJ, Angulo FJ, McLaughlin JM, et al. Impact and effectiveness of mRNA BNT162b2 vaccine against SARS-CoV-2 infections and COVID-19 cases, hospitalisations, and deaths following a nationwide vaccination campaign in Israel: an observational study using national surveillance data. *Lancet* 2021; **397**(10287): 1819-29.

14. Wang JG, Zhong ZJ, Mo YF, Wang LC, Chen R. Epidemiological features of coronavirus disease 2019 in children: a meta-analysis. *Eur Rev Med Pharmacol Sci* 2021; **25**(2): 1146-57.

15. Payne AB, Gilani Z, Godfred-Cato S, et al. Incidence of Multisystem Inflammatory Syndrome in Children Among US Persons Infected With SARS-CoV-2. *JAMA Netw Open* 2021; **4**(6): e2116420.

16. McCormick DW, Richardson LC, Young PR, et al. Deaths in Children and Adolescents Associated With COVID-19 and MIS-C in the United States. *Pediatrics* 2021.

17. Diaz GA, Parsons GT, Gering SK, Meier AR, Hutchinson IV, Robicsek A. Myocarditis and Pericarditis After Vaccination for COVID-19. *JAMA* 2021.

18. Shay DK, Shimabukuro TT, DeStefano F. Myocarditis Occurring After Immunization With mRNA-Based COVID-19 Vaccines. *JAMA Cardiol* 2021.

19. Menon P. New Zealand reports first death linked to Pfizer/BioNTech COVID-19 vaccine. Available at: [https://www.reuters.com/world/asia-pacific/new-zealand-reports-death-woman-after-pfizer-covid-vaccine-2021-08-30/](about:blank). Accessed 3 Sept 2021. 2021.

20. Team CC-R, Food, Drug A. Allergic Reactions Including Anaphylaxis After Receipt of the First Dose of Pfizer-BioNTech COVID-19 Vaccine - United States, December 14-23, 2020. *MMWR Morb Mortal Wkly Rep* 2021; **70**(2): 46-51.

21. Statistics ABo. Australia Bureau of Statistics. Vaccination numbers and statistics. Available at: [https://www.health.gov.au/initiatives-and-programs/covid-19-vaccines/numbers-statistics](about:blank). Accessed 28 Nov 2021.
